# Pro-smoking responses and attitudes due to point-of-sale tobacco displays in never smokers: A cross-sectional study in Hong Kong

**DOI:** 10.18332/tid/92585

**Published:** 2018-07-05

**Authors:** Yee Tak Derek Cheung, Sai Yin Ho, Man Ping Wang, Antonio Kwong, Vienna Lai, Tai Hing Lam

**Affiliations:** 1 School of Nursing, The University of Hong Kong, Hong Kong, China; 2 School of Public Health, The University of Hong Kong, Hong Kong, China; 3 The Hong Kong Council on Smoking and Health, Hong Kong, China

**Keywords:** point-of-sale, tobacco, advertisement, displays, never smokers

## Abstract

**INTRODUCTION:**

Never smokers’ responses to tobacco control policy are often overshadowed by the opposition from smokers and tobacco industry during policy advocacy and legislation. Very few studies have examined never smokers’ exposure to point-of-sale (POS) tobacco displays and their effects. Therefore, we investigated the exposure, pro-smoking responses due to and attitudes towards such displays in never smokers in Hong Kong.

**METHODS:**

We conducted two-stage, randomized cross-sectional telephone-based surveys in 2015 and 2016 of 1833 never-smoking adults. They were asked how often they noticed POS displays in the past 30 days (often, sometimes, never), whether they found POS displays attractive, felt encouraged to smoke, perceived POS displays as advertisements, and if they supported banning them. The distributions of the outcomes were analyzed by descriptive statistics with weighting to the general population. Risk ratios (RR) from Poisson regression models adjusted for sociodemographic characteristics were used to analyze the associations.

**RESULTS:**

Our results showed that, in never smokers, the younger were more likely to often notice POS displays (RR=0.80, 95% CI: 0.72–0.89, p<0.01). Finding POS displays attractive was associated with primary (RR=2.52, 95% CI: 1.51–4.22, p<0.01) and secondary education (RR=1.68, 95% CI: 1.16– 2.44, p=0.01) versus tertiary education. Often noticing displays was associated with perceived attractiveness (RR=1.90, 95% CI: 1.32–2.75, p<0.01). The positive association between often noticing displays and being encouraged to smoke was marginally significant (RR=4.05, 95% CI: 0.98–16.85, p=0.054). Respondents who often noticed POS displays (RR=0.87, 95% CI: 0.77–0.98, p=0.02) and did not perceive them as advertisements (RR=0.70, 95% CI: 0.61–0.98, p<0.01) showed less support on banning them than those who did not notice them.

**CONCLUSIONS:**

Frequent exposure to POS displays was associated with greater perceived attractiveness and lower support for banning them. A total ban on POS displays is needed to eliminate the advertising and normalization effect of POS displays.

## INTRODUCTION

Point-of-sale (POS) tobacco displays are used by tobacco companies to counter or circumvent tobacco advertising bans at retail outlets and in the mass media^1,2^. Tobacco companies pay retailers to establish fixtures that excessively display tobacco products at prominent positions and use audit programmes to monitor retailers’ compliance^1,2^. These POS displays have increased the adolescent smoking susceptibility and initiation, enhanced brand awareness^3-7^, and attracted smokers to buy tobacco products^8-10^, smoke^11-12^ and relapse^8,13,14^. Banning POS displays could effectively reduce recall and purchase of tobacco products in adult smokers and youth^15-19^. Despite the fact that 67 countries have banned POS direct advertisements, few (including Canada, New Zealand, Australia, UK, Norway and Thailand) have banned POS displays^20^.

Strong public support has been one of the determinants of the tobacco control advocacy in Hong Kong and elsewhere. Previous population surveys showed that there was strong public support for tobacco advertising bans, extension of statutory smoke-free areas and enlargement of pictorial health warnings^21-23^. These findings supported the Hong Kong Special Administrative Region (HKSAR) government to proceed with the making and implementation of various policies in the past 2 decades^24,25^. To understand how to raise the public awareness and support for banning POS displays, identifying the underlying factors of public support (or non-support) in the general population, including non-smokers, is critical. An observational study of adult smokers showed that exposure to POS displays led to lower support of banning them^26^, suggesting that POS displays are visual cues undermining public support to ban them. Previous qualitative studies have supported the notion that POS displays may normalize tobacco sale and use^8,27^, because POS displays are often placed at the eye level of cashiers in retail stores selling tobacco products, which are much more prominent and visible than other consumer products. Based on these findings, we proposed a ‘normalization’ hypothesis stating that members of the public who frequently see POS displays would get used to the presence of these displays and hence show lower support for banning them.

Our analysis was based on the combined datasets from the population-based telephone surveys conducted in 2015 and 2016 in Hong Kong, where all POS tobacco advertisements have been banned since 1999, but POS displays are still permitted. POS displays are present in almost all convenience stores and newspaper vendors, where tobacco product sale is the main source of income. The displays are either in the form of a transparent box displaying with a spot light and bright colours a few cigarette packs placed beside the cashier, or a prominent ‘powerwall’ showing a large number of packs of different brands and types behind the cashier. All customers can see the eye-catching POS displays when purchasing any goods or lining up at the cashier for other services. Despite Hong Kong having the lowest smoking prevalence of the developed world (10.1% in 2017)^28^ and being the most developed city in China, the HKSAR government has so far no proposal for banning these POS displays. The present study primarily examined the association between frequent exposure to POS displays and lower support for banning them. Our auxiliary analysis included never smokers’ pro-smoking responses due to the exposure to POS displays. We tested four hypotheses based on associations and outcome of frequently noticing POS displays as follows: 1) finding them attractive, 2) feeling being encouraged to smoke by them, 3) not perceiving them as advertisements, and 4) lower support for banning them.

## METHODS

### Study design and data collection

The Tobacco Control Policy-related Survey has been conducted annually by the Hong Kong Council on Smoking and Health (COSH) and the School of Public Health and School of Nursing of the University of Hong Kong (HKU) since 2013. The survey aimed to collect opinions about tobacco control in Hong Kong residents aged 15 years or older, including current smokers (who usually consumed at least 1 conventional cigarette per day), ex-smokers (who had used conventional cigarettes for at least 6 months in their lifetime but did not use any at the time of the survey) and never smokers. The present study was an analysis of the 2015 and 2016 telephone survey data^29^, which used two-stage random sampling and included questions about POS tobacco displays. Telephone numbers were drawn randomly from residential telephone directories and served as seed numbers from which another set of numbers were generated using the ‘plus/minus one/two’ method to capture unlisted numbers. When a target household was successfully contacted by phone, the household member whose next birthday was nearest to the interview date was selected out of all eligible and available household members and was invited to participate in the interview. Informed consent was obtained from every participant included in the study. After providing oral consent, the respondents completed a computer-based questionnaire administered by trained interviewers in either Cantonese or Putonghua. Each interview lasted 10 to 15 minutes. The survey was approved by the Institutional Review Board of the University of Hong Kong/Hospital Authority Hong Kong West Cluster (Ref: UW15–108/UW16–040). We followed the STROBE reporting guidelines in the study design and manuscript drafting.

The survey agent generated and dialed 149272 and 160050 telephone numbers in the 2015 and 2016 surveys, respectively. Of these, 59646 and 46109 were non-eligible numbers, and 60058 and 72072 were valid telephone numbers but the eligibility was not confirmed due to unanswered calls, incomplete screening, busy line, and non-Chinese speakers. In the 29568 and 41869 contacted households with a person eligible for the survey, 5252 (including 1834 never smokers) and 5151 respondents (including 1734 never smokers) consented participation and successfully completed the survey, with response rates of 17.7% and 12.3%, respectively. A large percentage of eligible participants (2015: 79.5%; 2016: 85.7%) consented and scheduled another time for the survey, but eventually did not complete it before the survey end-date, mainly because of the unavailability of the participants. As the survey period was limited, the survey agent needed to continuously draw new sample households to identify more eligible respondents. If we exclude these respondents, the response rate for the two survey years was 86.5% and 85.8%, respectively. Other eligible subjects were excluded due to incomplete surveys (1.5% and 1.2%, for 2015 and 2016, respectively), refusal (0.6% and 0.3%), or other reasons for loss of contact (0.7% and 0.5%). In all, 932 never smokers in 2015 and 901 in 2016 were randomly selected to answer the random question-set on POS tobacco displays.

All respondents answered the core questions and gave information on sex, age, education level, monthly household income and employment status, whereas random question sets were designed for randomly selected subsamples. Questions on POS displays were included in one of the random question sets in the 2015 and 2016 surveys. About half of the never smokers (2015: 932; 2016: 901) in both surveys were randomly selected to complete this question-set.

### Exposure and pro-smoking responses to POS displays

The respondents were asked: ‘In the past 30 days, how often have you noticed tobacco displays in retail shops selling tobacco products, such as convenience stores and newspaper vendors?’, with response options ‘Not noticing’, ‘Sometimes’, ‘Often’ and ‘Do not know, do not remember/refuse to answer (DK/RTA)’. ‘Not noticing’ and ‘Sometimes’ were combined as a reference group of the outcome ‘often noticing displays’ in the regression analysis. Perceived attractiveness of the tobacco displays was assessed by asking: ‘Do you think that POS displays are attractive?’, with response options ‘Very attractive’, ‘Attractive’, ‘Not attractive’, ‘Very unattractive’ and ‘DK/RTA’. Options were regrouped as ‘Attractive’ and ‘Unattractive’ in all the analysis. The question ‘Do you think that the POS displays have encouraged you to smoke?’ was asked with response options ‘Yes’, ‘No’ and ‘DK/RTA’.

### Perception towards POS displays

The respondents were asked: ‘Do you think POS displays are advertisements?’, with response options ‘Yes’, ‘No’ and ‘DK/RTA’.

### Support of banning POS displays

Finally, the respondents were asked if they supported banning POS displays. The response options were ‘Yes’, ‘No’ and ‘DK/RTA’.

### Statistical analysis

All analyses were adjusted using probability weights to match the distribution of sex and age of the Hong Kong general never-smoking population. The sex and age group distribution of Hong Kong’s mid-year never-smoking population in 2015 and 2016^30,31^, and the never-smoking prevalence in the 2015 Hong Kong Thematic Household Survey^32^ were used to construct the weight matrix. Cohen’s effect size (ω) was used to compare the distribution of sociodemographic characteristics between the weighted samples in 2015 and 2016.

The distributions of the variables were analyzed by descriptive statistics with weighting. Sensitivity analysis was conducted by excluding respondents who had not noticed the displays in the past 30 days. To reduce the width of the confidence intervals, we used Poisson regression models with robust error variance to assess whether sex, age group, marital status, education level, occupation and monthly household income were associated with often noticing displays^33^. As the prevalences of the key variables were greater than 10%, we estimated risk ratios (RR) instead of odds ratios. We then used similar modelling and included the frequency of noticing displays to examine the factors associated with other responses towards displays. Respondents who answered ‘DK/RTA’ were excluded from the regression models. All data analyses were conducted using STATA (Version 13, TX: StataCorp LP).

## RESULTS

Pooled analyses of never smokers in the 2015 and 2016 surveys showed that about 60% were females (Table 1); about 60% were married and 30% were single. Most had secondary or tertiary education (about 40% each). About half were employed. No significant difference in the sociodemographic characteristics, noticing POS displays, pro-smoking responses and attitudes between the two surveys were found (Supplementary Material 1).

**Table 1:** Responses and attitudes towards point-of-sale (POS) tobacco displays in never smokers (NS) (weighted %, 95% CI)

	*All NS % (95% CI)*	*Excluding NS not noticing % (95% CI)*
Noticed POS displays in the past 30 days	n=1706	
Not noticing	38.0 (35.6–40.5)	
Sometimes	30.5 (28.2–32.9)	
Often	31.5 (29.1–34.0)	
	n=1531	n=975
Found POS displays attractive	14.9 (13.1–16.9)	15.3 (13.0–17.9)
	n=1809	n=1007
Being encouraged to smoke by POS displays	1.8 (1.3–2.6)	2.1 (1.4–3.3)
	n=1711	n=995
Perceived POS displays as advertisements	70.0 (67.7–72.3)	67.5 (64.4–70.5)
	n=1731	n=976
Supported banning POS displays	62.8 (60.3–65.2)	59.2 (55.9–62.5)

NS: Never smokers, CI: confidence interval.

Responses of ‘don’t know’ or refusals to answer were excluded. All percentages were weighted by the age and sex distribution of the Hong Kong population (2015), and the smoking prevalence in the Hong Kong Thematic Household Survey (2015, Report No. 59)

About two-thirds of never smokers had noticed POS displays in the past 30 days, and about a third noticed them often (Table 1.) In all never smokers, about 14.9% found them attractive or very attractive. About 1.8% reported that they had been encouraged to smoke by them. Almost two-thirds (70.0%) perceived POS displays as advertisements. About 60% of all respondents supported banning them. The sensitivity analysis restricted to respondents who had seen POS displays in the past 30 days yielded similar results, suggesting that the response and support were not influenced by recent display exposure.

Table 2 shows that male (RR=1.23, 95% CI: 1.03–1.48, vs female), those aged 15–29 years (RR=2.39, 95% CI: 1.47–3.91, vs 60+), 30–39 years (RR=1.94, 95% CI: 1.30–2.88) and 40–49 years (RR=1.67, 95% CI: 1.15–2.44) were more likely to report often noticing POS displays. Younger respondents were more likely to report often noticing POS displays than older respondents, and the decreasing trend with increasing age was significant (RR=0.80, 95% CI: 0.72–0.89, p<0.01). Those with primary education, or below, reported less often noticing (RR=0.41, 95% CI: 0.23–0.73, p<0.01, vs tertiary), but greater perceived attractiveness (RR=2.52, 95% CI: 1.51–4.22, p<0.01). Often noticing displays (vs not noticing) was associated with finding them attractive (RR=1.90, 95% CI: 1.32–2.75, p<0.01), and lower support for banning displays (RR=0.87, 95% CI: 0.77–0.98, p=0.02) (Table 3), The effect size of the association between often noticing and being encouraged to smoke was large but was marginally significant (RR=4.05, 95% CI: 0.98–16.85, p=0.054). No association was found between often noticing displays and the perception that POS displays are advertisements (RR=0.99, 95% CI: 0.90–1.09, p=0.90), but not perceiving displays as advertisements was associated with lower support to ban them (RR=0.70, 95% CI: 0.61–0.98, p<0.01). Marital status, household income level and occupation were not associated factors of often noticing and pro-smoking responses at 5% significance level (not shown in the tables).

**Table 2:** Factors associated with: 1) often noticing POS displays, 2) finding POS displays attractive, 3) being encouraged to smoke by POS displays; from multivariate Poisson regression models

	*Often noticed POS displays in past 30 days (n=1315)*			*Found POS displays attractive (n=1133)*			*Being encouraged to smoke by POS displays (n=1300)*		

	*%*	*RR (95% CI)*	*p*	*%*	*RR (95% CI)*	*p*	*%*	*RR (95% CI)*	*p*
**Sex**									
Male	**37.2**	**1.23 (1.03–1.48)**	**0.02**	14.6	1.13 (0.80–1.60)	0.47	2.7	1.24 (0.47–3.25)	0.66
Female	27.9	1 (ref)		15.0	1 (ref)		1.2	1 (ref)	
**Age group, years**									
15–29	**45.7**	**2.39 (1.47–3.91)**	**<0.01**	7.8	0.78 (0.31–1.97)	0.60	1.7	3.77 (0.34–41.66)	0.28
30–39	**39.8**	**1.94 (1.30–2.88)**	**<0.01**	15.3	1.00 (0.52–1.91)	0.99	2.8	2.90 (0.27–30.92)	0.38
40–49	**34.9**	**1.67 (1.15–2.44)**	**0.01**	17.6	1.04 (0.58–1.85)	0.90	1.6	1.71 (0.2–14.79)	0.63
50–59	24.9	1.20 (0.83–1.75)	0.33	16.1	0.89 (0.52–1.51)	0.66	1.1	0.66 (0.05–9.00)	0.75
60 or above	14.9	1 (ref)	20.2		1 (ref)		2.0	1 (ref)	
**Education level**									
Primary or below	**10.1**	**0.41 (0.23–0.73)**	**<0.01**	**23.2**	**2.52 (1.51–4.22)**	**<0.01**	1.4	0.79 (0.09–7.32)	0.84
Secondary	28.7	0.87 (0.71–1.05)	0.14	**16.1**	**1.68 (1.16–2.44)**	**0.01**	1.7	1.04 (0.40–2.73)	0.94
Tertiary	40.7	1 (ref)		11.9	1 (ref)		2.0	1 (ref)	
**Noticed POS display in past 30 days**									
Not noticing				13.1	1 (ref)		1.0	1 (ref)	
Sometimes				12.1	1.15 (0.77–1.73)	0.49	1.4	1.74 (0.36–8.49)	0.49
Often				**18.3**	**1.90 (1.32–2.75)**	**<0.01**	2.9	3.98 (0.95–16.69)	0.054

Prevalence ratios in all models were adjusted for all sociodemographic variables, whether the respondent was living with smokers and survey year. Those with ‘don’t know’ or refusing to answer were excluded from the regression models. Estimates for marital status, household income level and occupation are not shown. All percentages were weighted by the age and sex distribution within each smoking status of the Hong Kong population (2015–2016).

**Table 3:** Factors associated with: 1) perceiving POS displays as advertisements, 2) support for banning POS displays

		*Perceiving POS displays as advertisements (n=1252)*			*Support for banning POS displays (n=1205)*	
	*%*	*RR (95% CI)*	*p*	*%*	*RR (95% CI)*	*p*
**Noticed POS displays in past 30 days**						
Not noticing	74.0	1 (ref)		67.7	1 (ref)	
Sometimes	65.8	0.92 (0.83–1.01)	0.08	61.8	0.91 (0.81–1.01)	0.09
Often	69.2	0.99 (0.90–1.09)	0.90	**56.7**	**0.87 (0.77–0.98)**	**0.02**
**Perceived POS displays as ads**						
Yes				68.8	1 (ref)	
No				**47.7**	**0.70 (0.61–0.98)**	**<0.01**

Prevalence ratios in all models were adjusted for all sociodemographic variables, whether the respondent was living with smokers, and survey year. Those with ‘don’t know’ or refusing to answer were excluded from the regression models. All percentages were weighted by the age and sex distribution within each smoking status of the Hong Kong population (2015–2016).

## DISCUSSION

This study is the first to show that often noticing POS displays was associated with lower support for banning displays in never smokers in Hong Kong. The findings supported the normalization hypothesis that displays are visual cues to increase acceptance and reduce support to ban them^26^. The potential explanation of this hypothesis is that when people get used to the presence of displays, they may perceive these displayed tobacco as ordinary consumer products rather than advertisements in the retail environment. Our findings showed that not perceiving POS displays as advertisements was associated with lower support for the display ban. However, we found no evidence to support that frequent exposure to POS displays was associated with the perception that POS displays are not advertisements. Another explanation of the normalization hypothesis is that while all other tobacco advertisements have been banned for many years (for at least 17 years in Hong Kong, for example), the fact that POS displays are permitted could mislead some people, especially those who often notice POS displays, to believe that POS displays are legitimate and acceptable. Our cross-sectional study may not be sufficient to confirm a causal relationship, but our findings are consistent with a qualitative study showing that POS displays lead adolescents to believe that many people smoke and that smoking is an acceptable behavior^6^. Our hypothesis that exposure to POS displays may reduce public support for banning POS displays warrants further study.

This study also revealed never-smoking adults’ exposure and pro-smoking responses due to POS displays. Often noticing POS displays was significantly associated with greater perceived attractiveness towards POS displays. The association between often noticing POS displays and being encouraged to smoke was marginally not significant due to the small absolute difference and low proportions of being encouraged to smoke (2.9% vs 1.0%). Yet, the large risk ratio with a small p-value suggests that the advertising effect of POS displays, in relation to measures of appeal and attractiveness, on never smokers was not negligible, particularly in Hong Kong where POS displays are present in nearly all convenience stores.

In never smokers, younger adults often noticed POS displays. This finding was consistent with previous studies, which showed that younger smokers were more aware of tobacco ads and POS displays^34-37^. Therefore, POS displays are effective channels to introduce tobacco products to young adults. However, younger age was not associated with more pro-smoking responses in never smokers, different from the smokers’ responses in previous studies^9,10,15,38^. As smoking mostly starts before adulthood^32^, the appealing effect of POS displays on non-smoking adults may be small. Another possible reason for the limited appealing effect is the mandatory graphic health warning that covers 50% of the cigarette packaging in Hong Kong. The POS advertising effect due to the attractive design of cigarette packs may be reduced when large pictorial warnings about tobacco’s health hazards are simultaneously shown. Further research is needed to evaluate this hypothesis.

Consistent with previous studies^9,35^, other socioeconomic characteristics, except education level, were not associated with noticing POS displays and pro-smoking responses. We found that never smokers with lower education level were less likely to be exposed to POS displays, but more likely to find POS displays attractive. Similar associations have been found in other studies on POS displays and other forms of tobacco advertising^34,38^. Never smokers with lower education level were more likely to be older adults, or people with less financial capacity to visit convenience stores very often, with possibly reduced exposure to POS displays. However, they may be less aware of the health hazards due to smoking than those with higher education^39^ and therefore more susceptible to environmental factors advocating smoking^40^.

Our study has several implications regarding tobacco POS displays in jurisdictions with or without POS advertising bans. First, we have shown that many never smokers perceived POS displays as tobacco advertisements hosted by the tobacco industry; this finding refutes the assertions that these displays are not advertisements and that they do not violate the law banning tobacco advertisement. Instead, POS displays can promote tobacco products and smoking behavior. Second, any exposure to POS displays in never smokers is unnecessary and may be harmful. Our study has extended the literature by showing that young never-smoking adults are more frequently exposed to these displays, and the displays are more appealing among less educated adults. Moreover, more exposure to POS displays was associated with lower support for a total ban. This finding implies that POS displays potentially undermine the public support for the total ban, and hence obstruct this tobacco control policy. Overall, Hong Kong’s incomplete policy of allowing POS displays has enabled the tobacco industry to use this loophole to establish aggressive advertising and normalize their products. To totally eliminate these negative influences, the ban on POS tobacco advertisements must include tobacco displays.

Our study had several limitations. First, as responses and attitudes towards POS displays might be influenced by other tobacco control measures and public education, our findings may not be fully applicable to places that substantially differ from Hong Kong. More similar studies in other countries with different tobacco control measures or different smoking prevalence are warranted. Second, our findings were from a cross-sectional study and thus cannot confirm whether the observed associations were causal. Yet, reverse causality in the current study (i.e. respondents who want to smoke may intend to notice POS displays more or show more pro-smoking responses) was not likely, because the respondents were never smokers who normally had no intention to smoke or buy tobacco products. Third, the frequency of seeing the displays could only be measured in subjective categories (e.g. often and occasionally) and by self-reporting. The accuracy of self-reporting, and of the recalled responses to POS tobacco displays, may be lower than that of advanced real-time measurements, such as ecological momentary assessments. Nevertheless, we used this research design as most previous studies in this field did to improve comparability. Lastly, many eligible participants consented and scheduled another time for the telephone interview, but the interviews were not completed before the end of the survey period. The low response rate might affect the external validity of the findings.

## CONCLUSIONS

In Hong Kong, where POS tobacco displays were (and still are) not banned, many never smokers noticed them and perceived them as advertisements. Frequent exposure to POS displays was associated with greater perceived attractiveness and lower support for banning them. All these findings support the hypothesis that the displays could promote smoking in never smokers and could become normalized when they are seen more frequently. A total ban on POS tobacco display is needed to eliminate the advertising and normalization effect of POS displays.
