# Supplementary material for: Pro-smoking responses and attitudes due to point-of-sale tobacco displays in never smokers: A cross-sectional study in Hong Kong
Source: Tob Induc Dis. 2018 Jul 5;16:32. doi: 10.18332/tid/92585 (PMC6659512; doi:10.18332/tid/92585)
Supplement: Supplementary file 1 [file TID-16-32-s1.pdf]

Supplementary Material 1. Weighted proportion of respondent characteristics by survey year

		2015<br>n=932	2016<br>N=901	Cohen's ω
**Sex**	Male	38.4	38.2	<0.01
	Female	61.6	61.8	
**Age (years)**	15–29	20.5	21.5	0.07
	30–39	17.8	17.6	
	40–49	18.1	16.8	
	50–59	20.6	18.9	
	60+	22.3	24.2	
	DK/RTA	0.7	1.0	
**Marital status**	Single	30.4	30.7	0.04
	Married	61.7	59.5	
	Divorced/Widowed	6.5	8.5	
	DK/RTA	1.4	1.2	
**Education level**	Primary or below	11.7	11.7	0.08
	Secondary	46.4	44.9	
	Tertiary or above	41.4	42.4	
	DK/RTA	0.6	1.0	
**Employment status**	Employed	54.3	41.7	0.26
	Full-time student	12.2	13.8	
	Home-maker	15.3	20.9	
	Unemployed	1.8	3.0	
	Retired	16.0	19.6	
	DK/RTA	0.5	1.1	
**Monthly household income (HK$) (US$1= HK$7.8)**	<10000	12.3	12.2	0.27
	10000–19999	14.3	14.1	
	20000–29999	11.8	18.4	
	30000–39999	11.6	10.2	
	≥40000	24.8	28.2	
	DK/RTA	25.3	16.9	
**Living with smokers**	Yes	15.9	11.8	0.13
	No	84.1	88.2	
**Noticing the POS displays**	Never	34.0	37.2	0.17
	Occasion	31.9	25.0	
	Always	27.2	31.7	
	DK/RTA	7.0	6.1	
**Perceived the displays attractive**	Very attractive	2.5	2.4	0.15
	Attractive	10.0	10.5	
	Unattractive	47.9	44.6	
	Very unattractive	23.3	29.4	
	DK/RTA	16.4	13.1	

**Being encouraged to smoke by the displays**	Yes<br>No<br>DK/RTA	2.2<br>96.2<br>1.6	1.3<br>97.9<br>0.8	0.11
**Perceived the displays as ads**	Yes<br>No<br>DK/RTA	62.6<br>30.7<br>6.7	69.8<br>25.7<br>4.4	0.17
**Support to ban the displays**	Yes<br>No<br>DK/RTA	57.9<br>35.6<br>6.5	61.2<br>34.9<br>3.9	0.14

All descriptive values are column percentages, and the values from the survey were weighted by the age and sex distribution of the Hong Kong population (2015 & 2016), and the smoking prevalence in the Hong Kong Thematic Household Survey (2015, Report No. 59). Cohen's effect size (ω) was used to compare the distribution of sex, age, marital status and smoking status of our weighted sample to the general population.
DK: Don't know, RTA: Refused to answer.
[1]Source: Hong Kong Monthly Digest of Statistics November 2015 and Population and Household Statistics Analyzed by District Council District 2015.
[2] Effect size comparing the overall distribution in the sample with the HK Census.
[3]No population-based data were available from the Hong Kong Census.
[4]Employed and retired were combined as one group in the Hong Kong Census.
